# Factors Affecting Death and Severe Injury in Child Motor Vehicle Passengers

**DOI:** 10.3390/healthcare9111431

**Published:** 2021-10-24

**Authors:** Wataru Ishii, Masahito Hitosugi, Mineko Baba, Kenji Kandori, Yusuke Arai

**Affiliations:** 1 Kyoto Daini Red Cross Hospital, Critical Care Center, Emergency of Medicine, Haruobi, Kamazamarutamachi, Kamigyo, Kyoto 602-8026, Japan; knj.kandori@gmail.com (K.K.); sndlwd_1@yahoo.co.jp (Y.A.); 2 Department of Legal Medicine, Shiga University of Medical Science, Tsukinowa, Seta, Otsu, Shiga 520-2192, Japan; hitosugi@belle.shiga-med.ac.jp; 3 Center for Integrated Medical Research, Keio University School of Medicine, Tokyo 160-8582, Japan; mineko@keio.jp

**Keywords:** child, motor vehicle passenger, traffic accident, Japan Trauma Data Bank

## Abstract

Saving children from motor vehicle collisions is a high priority because the injury rate among motor vehicle passengers has been increasing in Japan. This study aimed to examine the factors that influence death and serious injury in child motor vehicle passengers to establish effective preventive measures. To identify these factors, we performed a retrospective study using a nationwide medical database. The data of child motor vehicle passengers younger than 15 years (*n* = 1084) were obtained from the Japanese Trauma Data Bank, registered from 2004 to 2019. Physiological variables, outcomes, and injury severity were compared between fatal and non-fatal patients and between those with and without severe injuries. Multivariate logistic regression analysis was performed to determine factors affecting fatality and severe injury. The Glasgow Coma Scale score (odds ratio (OR): 1.964), body temperature (OR: 2.578), and the Abbreviated Injury Scale score of the head (OR: 0.287) were identified as independent predictors of a non-fatal outcome. Systolic blood pressure (OR: 1.012), the Glasgow Coma Scale score (OR: 0.705), and Focused Assessment with Sonography for Trauma positivity (OR: 3.236) were identified as independent predictors of having severe injury. Decreasing the severity of head injury is the highest priority for child motor vehicle passengers to prevent fatality and severe injury.

## 1. Introduction

Death and injury resulting from motor vehicle collision (MVC) remain serious problems globally. The number of deaths due to MVCs has been increasing and reached 1.35 million worldwide in 2016. However, the rate of death due to MVCs relative to the size of the world’s population has stabilized in recent years [1]. The burden of injury and death from MVCs is disproportionately borne by vulnerable road users such as children. With the improvement of vehicle safety equipment, laws of seat belt use, improvement of pre-hospital and trauma care, and education on trauma critical care, the mortality rate of motor vehicle passengers in MVCs has been decreasing in Japan [2,3,4,5]. However, MVC injury is currently the leading cause of death in children and young adults aged 5 to 29 years [1]. This finding indicates a need for a shift in the current child health agenda, which has largely neglected road safety.

In Japan, the population aged 0 to 5 years (pre-school children) decreased from 8.22 million in 1989 to 5.73 million in 2019, and the population aged 6 to 11 years (elementary school children) decreased from 9.47 million to 6.27 million [6]. This low birth rate is considered a large nationwide problem. In 2020, the number of births was 841,000 and the number of children younger than 15 years was 1.51 million, which are the lowest reported numbers [7]. Therefore, saving a child from unintentional injuries, especially in MVCs, is a large challenge in Japan. 

The Japanese government established the Traffic Safety Measures Basic Law in 1970 [8]. With this law, the Traffic Safety Basic Plan specified goals every 5 years after 1971, and traffic safety measures were comprehensively and systemically promoted. An essential goal is to achieve a society with no MVCs. In 2021, the Japanese government established a new set of objectives to reduce the number of fatalities to fewer than 2000 and to reduce the number of severely injured people to fewer than 22,000 by 2025 [9]. Furthermore, under progression of a declining birth rate, an improvement in the environment in which children can safely move and grow up was also promoted. To achieve these goals, health professionals need to consider effective measures to prevent fatality in children due to MVCs. According to recent statistics of MVCs in Japan, the rate of vehicle passenger injuries has dramatically increased in 10 years (10.6% in 1990 to 34.5% in 2019) [10]. Therefore, there needs to be a focus on preventing fatalities in child vehicle passengers. Previous studies investigated the factors affecting the severity of injury in child passengers using police data or a crash database with collision information [11,12,13,14,15]. Kahani et al. reported that the severity of injuries was related to the use of seat belts and the cause and the form of the crash [11]. Chang et al. showed that the severity of MVC injuries depended on the vehicle type, and Yan et al. found that rear-end collisions were more likely to occur at a higher speed limit, at nighttime, and with a wet road surface [12,13]. Durbin et al. reported the use of correct in-vehicle restraints and child restraints is necessary to reduce the cause of death in MVCs in children [14]. Another study reported that the severity of injuries in children riding in a vehicle increases according to the passenger compartment intrusion [15]. These above-mentioned factors included the type of vehicle involved, the situation of the collision, major injuries of the victim, and other associated factors. However, no studies have examined the factors affecting the fatality of child motor vehicle passengers based on nationwide medical data obtained at a hospital.

Therefore, the main objective of this study was to examine the factors affecting fatality and severe injury in child vehicle passengers using a national trauma database to establish effective preventive measures.

## 2. Materials and Methods

### 2.1. Study Design and Patient Selection

This observational study was a retrospective analysis of data from a national hospital-based database called the Japanese Trauma Data Bank (JTDB). The JTDB is a nationwide trauma registry in Japan that contains data recorded since 2003 by the Japanese Association for the Surgery of Trauma and the Japanese Association for Acute Medicine. This registry is similar to trauma databases in North America, Europe, and Oceania [16,17,18,19]. Approximately 372,000 patients with trauma were registered until 2019 [16]. A total of 55 hospitals participated in the registry in 2005, and more than 288 hospitals participated in the registry in 2019; this accounted for approximately 75% of the critical care centers in Japan. The inclusion criteria for the JTDB were patients with trauma who had any injury with an Abbreviated Injury Scale (AIS) score of ≥3. The JTDB collected information on the manners and mechanisms of injuries, vital signs, anatomical and physiological injury severity, pre-hospital and in-hospital treatment, and outcome.

**Figure 1:**
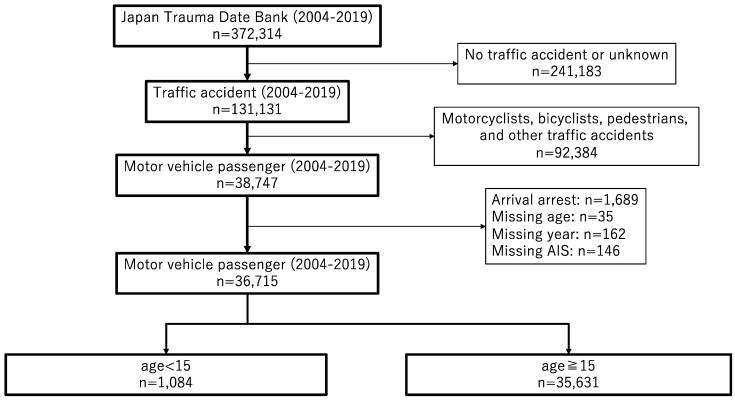
Flowchart of patient enrollment.

Data were obtained from the JTDB in December 2020. A total of 372,314 patients were registered in the JTDB from 2004 to 2019. Of these, 38,747 were MVC-related trauma patients. Cases were excluded if the patient arrived with cardiopulmonary arrest (*n* = 1689), if the age, accident year, or AIS score was unclear, or there were missing data (*n* = 343). The data from 36,715 vehicle passengers (including drivers) were assessed. Among them, patients younger than 15 years were selected. Finally, 1084 patients were selected for analysis (Figure 1).

The following information was obtained for each patient: age, sex, vehicle seating position, the duration between an emergency call and hospital arrival, vital signs on arrival to the hospital, including systolic and diastolic blood pressure (s-BP and d-BP), heart rate (HR), respiration rate (RR), body temperature (BT), Glasgow Coma Scale (GCS) score, Focused Assessment with Sonography for Trauma (FAST) examination results, AIS score (version 1998) for each body region, Injury Severity Score (ISS), Revised Trauma Score (RTS), Trauma and Injury Severity Score Probability of Survival (TRISSPs), and hospital discharge outcome. The AIS score is used to categorize the injury type and severity anatomically in each body region on a scale from 1 (minor) to 6 (clinically untreatable). The ISS, which is useful for assessing the severity of multiple injuries, is the sum of the squares of the highest AIS score in each of the three most severely injured body regions. The RTS is a method of assessing severity on the basis of physiological indices, where the best is 7.84 points and the most severe is 0 points. The TRISSPs adds physiological and anatomical severity and age factors to calculate the predicted survival rate. Death with a probability of survival >0.5 is considered an avoidable death, ≥0.25 and ≤0.5 is considered a preventable trauma death, and <0.25 is considered a non-preventable death.

### 2.2. Statistical Analysis

Categorical variables are shown as the proportion or frequency. Continuous variables are shown as mean ± standard deviation for values that followed a normal distribution and as the median and interquartile range (IQR) for values that were not normally distributed. The chi-square test was used to compare the prevalence between two groups. To identify differences in values between two groups, the Student’s t-test was used for values with a normal distribution, while the Wilcoxon test was used for values without a normal distribution. A *p* value of ≤0.05 was considered statistically significant. Logistic regression analyses were performed to identify which variables were independently associated with a poor outcome or having severe injury. The analyses were performed with SPSS Ver. 23.

### 2.3. Endpoint

Significant factors affecting death or severe injuries in child motor vehicle passengers were examined using data from the JTDB.

## 3. Results

### 3.1. Comparison of Patients’ Outcomes 

A total of 29 (3.2%) patients died (fatal group) and 875 survived (non-fatal group). The patients’ background characteristics and vital signs at the time of hospital arrival were compared between the two groups (Table 1). The fatal group had a significantly lower s-BP, d-BP, BT, RR, and GCS score, and a significantly higher HR at hospital arrival compared with those in the non-fatal group. Regarding injury severity, the ISS and AIS of the head, chest, and spine were significantly higher, but AIS of face was significantly lower in the fatal group than in the non-fatal group (Table 2). The RTS and TRISSPs were significantly lower in the fatal group than in the non-fatal group.

### 3.2. Comparison of Patients with and without Severe Injuries

A total of 651 (60.1%) patients had severe injuries with a maximum AIS score ≥3 and 433 did not have severe injuries (maximum AIS score < 3). Patient background characteristics and vital signs at the time of hospital arrival were compared between patients with and without severe injuries (AIS ≥ 3) (Table 3). There were no significant differences in the background characteristics between the two groups of patients. At the hospital, patients with severe injuries had a significantly lower GCS score and a significantly higher s-BP, HR, RR, FAST positive rate, and mortality rate than those of patients without severe injuries. The RTS and TRISSPs were significantly lower, and the ISS was significantly higher in patients with severe injuries than in those without severe injuries (Table 4). 

### 3.3. Factors Affecting the Outcome

To identify variables that were independently associated with fatality, logistic regression analyses were performed on the basis of the comparison results. Because significant differences were found in the comparisons for age, s-BP, d-BP, HR, RR, BT, the GCS score, and AIS scores of the head, face, chest, and spine, these variables were included in the logistic regression analysis. The GCS score (odds ratio (OR): 1.964), BT (OR: 2.578), and the AIS score of the head (OR: 0.287) were identified as independent predictors of a non-fatal outcome (Table 5). 

### 3.4. Factors Affecting Severe Injuries

To identify variables that were independently associated with having severe injuries, logistic regression analyses were performed on the basis of the comparison results. Significant differences were found in the comparisons for s-BP, HR, RR, the GCS score, FAST results, and the mortality rate. Therefore, these variables were included in the logistic regression analysis. We found that s-BP (OR: 1.012), the GCS score (OR: 0.705), and FAST positivity (OR: 3.236) were independent predictors of having severe injuries (Table 6).

## 4. Discussion

Child safety in traffic situations has been considered, especially for pedestrians. Although road safety interventions for children are effective in increasing knowledge about safety, they do not improve traffic behavior [20]. Studies have suggested that speed control through active measures has the greatest benefit, and education and training programs for altering pedestrian behavior on the road have the least benefits based on pedestrian behavior (e.g., gap acceptance, preference of route choice, and location for crossing the road) [21]. Children have a limited sensory or cognitive ability to cope with modern traffic. Therefore, there are many environmental elements and factors related to road traffic that must be improved to achieve safety perception for children [22]. Additionally, environmental factors, such as whether the area is urban, and road conditions and traffic volume, contribute to the severity of injury in child vehicle passengers [23,24]. Therefore, to improve children’s safety in traffic, there needs to be a focus not only on road safety programs for children, but also on improving environmental elements and on adult supervision of children’s movements. 

Monitoring trends and trauma outcomes can assist in identifying effective injury prevention strategies and treatment. The JTDB registers data of patients with injuries and records pre-hospitalization and hospital-related information, including the clinical outcome. Therefore, analyses using this database can provide useful evidence for prevention of injury. Two previous reports used the JTDB for investigating child injuries [25,26]. One of these studies focused on child injuries due to MVCs and described the injury patterns and outcomes relating to the seating position of the vehicle. This previous study suggested that rear seating was associated with a higher incidence of head and chest injuries for victims aged 6 to 12 years. [26]. However, no studies have investigated the factors that independently affect fatality in child motor vehicle passengers involved in a collision. 

The World Health Organization classifies traumatic brain injury as the leading cause of death and disability in children and young adults worldwide, and traumatic brain injury is the largest injury-related factor [27]. The present study showed that a lower GCS score was significantly related to fatality and injury with a maximum AIS score ≥3, and a higher head AIS score was significantly related to fatality. Traumatic head injury is the leading cause of mortality and disability in children and adolescents [28,29,30,31]. A retrospective study in Australia showed that having a head injury and greater injury severity were associated with a higher mortality rate [32]. A study of a trauma registry on children younger than 15 years in New South Wales showed that a high ISS and injured body region of head or neck were predictors of in-hospital mortality [33]. The GCS is a scoring system describing the state of consciousness and is also used for quantifiable determination of traumatic brain-injured patients’ prognoses. For road traffic injuries, higher values of the GCS score positively reflect the time to recovery [34]. Therefore, for preventing fatality due to MVCs in child passengers, decreasing the severity of head injury and maintaining a high GCS score are considered as the highest priority. To achieve this objective, the use of restraints that are appropriate for the child’s age is recommended. Some reports have indicated that the correct use of restraints and seat belts reduces the risk of injury by 71% in children, the risk of serious injury by 45% in those aged 4 to 8 years, and the risk of serious injury or death by up to 50% in older children [35]. However, because some countries have a low restraint-use rate in children, implementing comprehensive legislation for restraint use in children should be promoted, especially in developing countries [36].

The adult brain is thought to have less physiological and neuroanatomical reorganization following injury [37,38]. A previous study compared functional outcomes at discharge following traumatic brain injury in children versus adults and showed a three times higher chance of good functional outcome at the time of discharge in children compared with adults [39]. Other studies have also suggested improved functional outcomes in the pediatric population following traumatic brain injury [40,41]. Another report suggested that younger people tolerated longer periods of coma or decerebration better than older people [40]. The reasons why children have a better functional outcome than adults following traumatic brain injury are considered as follows: children have greater flexibility of cranial bones, which may absorb traumatic forces, thereby reducing focal brain injury [42]. In children, the central nervous system retains the ability to recover and adapt secondary compensatory mechanisms following injury [43]. The ability of neuronal circuits in children enables structural and functional adaptive changes [44]. Therefore, especially for children, preventive measures for reducing mortality with an increase in the GCS score or a decrease in the head ISS may also contribute to an improvement of functional outcomes. 

A previous study on adult vehicle passengers aged ≥ 15 years investigated factors affecting fatality using a similar database to the JTDB. Between 2016 and 2017, the factors of male sex (OR: 2.49), being a front-seat passenger (OR: 1.99), age (OR: 1.05), the GCS score (OR: 0.77), the AIS score of the abdomen (OR: 1.40), and FAST positivity (OR: 2.40) were identified as independent predictors of fatality [5]. In the present study, a similar trend was found for the GCS score, but there were some differences compared with this other study. In adults, FAST positivity and a higher AIS score of the abdomen suggest that intra-abdominal injuries affect fatality. This finding is due to the fact that adult vehicle passengers with severe injuries can easily receive external forces via the seatbelt, steering wheel, or other interior structures of the vehicle on the abdomen in the upright position. However, in children, because of using child restraints or the larger proportion of the head to the trunk, passengers might suffer more from head injuries than abdominal injuries. Therefore, the AIS score of the abdomen and FAST positivity were not determined as significant factors for affecting fatality in our study. However, FAST positivity was an independent factor for having injuries with an AIS score ≥ 3. Therefore, although our finding suggested that intra-abdominal injuries were not a significant factor for fatality, they were an important factor for suffering from severe injuries. In this study, the seating position was not a significant factor in contrast to the previous study on adults. This difference between studies is likely because of the difference in the distribution of the seating position between children and adults. Most child passengers sat in the rear seat in the present study, whereas more than three-quarters of adult vehicle passengers sat in the driver’s seat in the previous study [5].

With regard to vital signs in passengers, our results suggest that a lower BT was significantly associated with the fatality rate. This finding is similar to that found in adults who were examined between 2004 and 2008 [5]. Therefore, measuring BT in patients would be useful for estimating the prognosis. However, because the standard values of BT in children are different from those in adults, medical staff need to understand standard absolute values in each age group (i.e., infants, schoolchildren, or adolescents) at evaluation. Additionally, s-BP was a significant factor for having injuries with an AIS score ≥ 3 in our study. However, the OR was low (1.012), and this factor might not overcome the other factors described above.

## 5. Conclusions

To prevent fatal or severe injuries in child motor vehicle passengers, prevention of head injuries, with a reduction in the AIS score of the head and elevation of the GCS score, is a high priority. Additionally, hypothermia at hospital admission is a prognostic factor and active medical intervention is considered necessary.

## 6. Limitation

This study has some limitations. First, information on crashes, such as collision details (type of car, collision direction, and velocity), seat belt use, child restraints use, and airbag deployment, were not present in the JTDB registry. In Japan, the use of child restraints is legally required for child passengers aged ≤ 6 years. Generally, during an MVC, age- and size-appropriate restraint use is the most effective way to prevent injury and death [45]. The proper use of restraints has reduced the risk of injury and fatality in child passengers and using restraints corresponding to age is important for child safety [46,47,48]. Moreover, proper use of a booster seat and seat belt use among children in the rear seat reduced the hospital cost [49]. Furthermore, there are urban–rural differences regarding restraint use. Parents in urban areas may not use children’s restraints appropriately when taking short trips, while they may use them appropriately for interurban travel. This is an issue that needs to be resolved in future studies. Therefore, future studies should examine the associations between accident data collected by the police department, some environmental data, and medical data based on hospital records in Japan. 

Second, this study did not include all injured vehicle passengers in Japan. However, the JTDB represents trauma cases to a similar degree to that in databases in North America, Europe, and Oceania. Additionally, almost all certificated trauma educational institutions and many critical care centers participated in this database. The JTDB is the only prospective, nationwide, hospital-based trauma registry in Japan. Therefore, we believe that our analyses provided representative results. Third, because this database was hospital-based, cases of instant death pronounced at the collision scene were not included. Furthermore, patients who experienced cardiopulmonary arrest on arrival were excluded from this study. In these patients, the physiological parameters and details of the injuries were not obtained because of the lack of examinations. Therefore, including these out-of-hospital deaths would likely not improve the reliability of the present analyses.

## Figures and Tables

**Table 1:** Comparison of patients’ characteristics and data at hospital arrival between those aged < 15 years in the fatal and non-fatal groups (2004–2019). FAST: Focused Assessment with Sonography for Trauma.

	Non-Fatal	Fatal	
	(*n* = 875)	(*n* = 29)	*p* Value
Age (years)	6.6 ± 4.1	3.4 ± 4.2	<0.0001
Sex, n (%)			0.2412
Male	54.5	65.5	
Female	45.5	34.5	
Seating position (%)			0.7982
Front seat passenger	26.3	24.1	
Rear seat passenger	73.7	75.9	
Systolic blood pressure (mmHg)	116.4 ± 20.1	85.1 ± 41.3	<0.001
Diastolic blood pressure (mmHg)	69.1 ± 16.2	55.0 ± 28.8	<0.001
Heart rate (beats/min)	113.9 ± 28.8	120.4 ± 46.9	0.026
Respiration rate (breaths/min)	25.9 ± 9.7	19.5 ± 16.9	0.001
Body temperature (°C)	36.8 ± 0.8	34.8 ± 1.8	<0.001
Glasgow coma scale	13.2 ± 3.1	4.3 ± 1.7	<0.001
FAST positive (%)	11.5	10.0	0.833

**Table 2:** Comparison of the Abbreviated Injury Scale (AIS) score by body region, Injury Severity Score (ISS), Revised Trauma Score (RTS), and Trauma and Injury Severity Score Probability of Survival (TRISSPs) in patients aged < 15 years between the fatal and non-fatal groups (2004–2019). * The median value in the non-fatal group was significantly higher than that in the fatal group. ** The median value in the non-fatal group was significantly lower than that in the fatal group.

	Non-Fatal	Fatal	
	(*n* = 875)	(*n* = 29)	*p* Value
AIS, median (IQR)			
Head **	1.0 (0.0–3.0)	1.0 (0.0–3.0)	<0.001
Face *	0.0 (0.0–0.0)	0.0 (0.0–0.0)	<0.001
Neck	0.0 (0.0–1.0)	0.0 (0.0–1.0)	0.912
Chest **	0.0 (0.0–0.0)	0.0 (0.0–0.0)	0.002
Abdomen	0.0 (0.0–0.0)	0.0 (0.0–0.0)	0.584
Spine **	0.0 (0.0–0.0)	0.0 (0.0–0.0)	0.014
Upper extremities	0.0 (0.0–0.0)	0.0 (0.0–0.0)	0.093
Lower extremities	0.0 (0.0–1.0)	0.0 (0.0–1.0)	0.085
ISS, median (IQR) **	10.0 (5.0–17.0)	25.5 (25.0–10.25)	<0.001
RTS, median (IQR) *	7.84 (6.90–7.84)	3.80 (1.53–4.84)	<0.001
TRISSPs, median (IQR) *	0.99 (0.97–0.99)	0.54 (0.15–0.82)	<0.001

**Table 3:** Comparison of patients’ characteristics and data at hospital arrival in those aged < 15 years with an AIS score ≥3 and those with an AIS score < 3 (2004–2019). FAST: Focused Assessment with Sonography for Trauma.

	Age < 15 (2004 to 2019)	Age < 15 (2004 to 2019)	
	AIS ≥ 3	AIS < 3	
	(*n* = 651)	(*n* = 433)	*p*-Value
Age (years)	6.4 ± 4.3	6.3 ± 4.0	0.694
Sex, n (%)			0.544
Male	56.4	54.5	
Female	43.6	45.5	
Seating position (%)			0.111
Front seat passenger	25.2	29.7	
Rear seat passenger	74.8	70.3	
Systolic blood pressure (mmHg)	116.1 ± 23.5	111.7 ± 19.7	0.002
Diastolic blood pressure (mmHg)	68.8 ± 18.0	67.6 ± 12.9	0.268
Heart rate (beats/min)	116.7 ± 30.8	109.3 ± 27.3	<0.001
Respiration rate (breaths/min)	26.2 ± 10.6	24.2 ± 9.1	0.003
Body temperature (°C)	36.7 ± 1.0	36.8 ± 0.7	0.180
Glasgow coma scale	12.2 ± 3.8	14.5 ± 1.5	<0.001
FAST positive (%)	13.7	4.9	<0.001
Death rate (%)	4.5	0.4	0.001

**Table 4:** Comparison of the ISS, RTS, and TRISSPS in patients aged < 15 years between those with an AIS score ≥ 3 and those with an AIS score < 3 (2004–2019). * The median value for an AIS score ≥ 3 was significantly higher than that for an AIS score < 3. ** The median value for an AIS score ≥ 3 was significantly lower than that for an AIS score < 3.

	Age < 15 (2004 to 2019)	Age < 15 (2004 to 2019)	
	AIS ≥ 3	AIS < 3	
	(*n* = 651)	(*n* = 433)	*p* Value
ISS, median (IQR) *	16.0 (10.0–22.0)	1.0 (1.0–4.0)	<0.001
RTS, median (IQR) **	7.55 (6.61–7.84)	7.84 (7.55–7.84)	<0.001
TRISSPs, median (IQR) **	0.99 (0.96–0.99)	1.00 (1.00–1.00)	<0.001

**Table 5:** Results of multivariate analyses for influencing death of the age group of <15 years. OR: odds ratio, 95%; CI: 95% confidence interval.

	(*n* = 904)			
	OR	Lower 95% CI	Upper 95% CI	*p*-Value
Max AIS Head	0.287	0.116	0.714	0.007
GCS	1.964	1.291	2.988	0.002
BT	2.578	1.228	5.415	0.012

**Table 6:** Results of multivariate analyses for influencing severity injury of the age group of <15 years with severe injuries. OR: odds ratio, 95%; CI: 95% confidence interval.

	(*n* = 1084)			
	OR	Lower 95% CI	Upper 95% CI	*p*-Value
sBP	1.012	1.002	1.022	0.016
GCS	0.705	0.636	0.783	<0.001
FAST	3.236	1.631	6.423	<0.001
